# Host Biomarkers for Distinguishing Bacterial from Non-Bacterial Causes of Acute Febrile Illness: A Comprehensive Review

**DOI:** 10.1371/journal.pone.0160278

**Published:** 2016-08-03

**Authors:** Anokhi J. Kapasi, Sabine Dittrich, Iveth J. González, Timothy C. Rodwell

**Affiliations:** Foundation for Innovative New Diagnostics (FIND), Campus Biotech Building B2 Level 0, 9 Chemin des Mines, 1202, Geneva, Switzerland; Universidade Nova de Lisboa Instituto de Higiene e Medicina Tropical, PORTUGAL

## Abstract

**Background:**

In resource limited settings acute febrile illnesses are often treated empirically due to a lack of reliable, rapid point-of-care diagnostics. This contributes to the indiscriminate use of antimicrobial drugs and poor treatment outcomes. The aim of this comprehensive review was to summarize the diagnostic performance of host biomarkers capable of differentiating bacterial from non-bacterial infections to guide the use of antibiotics.

**Methods:**

Online databases of published literature were searched from January 2010 through April 2015. English language studies that evaluated the performance of one or more host biomarker in differentiating bacterial from non-bacterial infection in patients were included. Key information extracted included author information, study methods, population, pathogens, clinical information, and biomarker performance data. Study quality was assessed using a combination of validated criteria from the QUADAS and Lijmer checklists. Biomarkers were categorized as hematologic factors, inflammatory molecules, cytokines, cell surface or metabolic markers, other host biomarkers, host transcripts, clinical biometrics, and combinations of markers.

**Findings:**

Of the 193 citations identified, 59 studies that evaluated over 112 host biomarkers were selected. Most studies involved patient populations from high-income countries, while 19% involved populations from low- and middle-income countries. The most frequently evaluated host biomarkers were C-reactive protein (61%), white blood cell count (44%) and procalcitonin (34%). Study quality scores ranged from 23.1% to 92.3%. There were 9 high performance host biomarkers or combinations, with sensitivity and specificity of ≥85% or either sensitivity or specificity was reported to be 100%. Five host biomarkers were considered weak markers as they lacked statistically significant performance in discriminating between bacterial and non-bacterial infections.

**Discussion:**

This manuscript provides a summary of host biomarkers to differentiate bacterial from non-bacterial infections in patients with acute febrile illness. Findings provide a basis for prioritizing efforts for further research, assay development and eventual commercialization of rapid point-of-care tests to guide use of antimicrobials. This review also highlights gaps in current knowledge that should be addressed to further improve management of febrile patients.

## Introduction

Acute febrile illness (AFI) is one of the most common reasons for seeking medical care in any region of the world and it refers to sudden onset of fever, typically at least 38°C along with symptoms such as headache, chills or muscle and joint pains [1]. Although there are both infectious and noninfectious causes for febrile illness, AFI most often follows infection by a wide diversity of pathogens such as bacteria, viruses, fungi, and parasites [2], and is a major cause of morbidity and mortality, especially in children. The incidence and etiology of infectious causes of febrile illness varies geographically, seasonally, and by human immunodeficiency virus (HIV) prevalence and other comorbidities, and has also been shifting with the widespread use of effective vaccines against causative organisms, environmental changes and economic development [3, 4].

Febrile illnesses are often treated empirically without determining the etiology. In 2010, the World Health Organization (WHO) recommended that all patients presenting with AFI in malaria-endemic countries should be tested for malaria before treatment [5]. This was implemented as an effort to reduce unnecessary use of antimalarial drugs and also to enable non-malaria febrile patients to receive the appropriate care and treatment in a timely manner [2, 6]. Results from studies on malaria rapid diagnostic tests have shown that up to 80% of febrile illness, even in malaria-endemic regions, is caused by other pathogens [6–9]. Many of these infections can be treated with targeted therapy, but without confirmatory tests, they are clinically indistinguishable from other infections even with clinical history and physical examination information.

In many malaria-endemic countries, no reliable incidence data on other causes of febrile illness exist [6, 8, 10, 11]. Unfortunately, in the absence of additional information, most clinicians assume that a non-malarial febrile illness is most likely a bacterial infection, leading to indiscriminate use of antibiotics [12]. This is partially due to the absence of accurate diagnostic tools to guide selection of appropriate therapies and particularly in rural and resource-limited regions, where there may be limited capacity for conventional laboratory diagnostic testing [13]. This lack of evidence-based treatment is contributing to the global antimicrobial resistance (AMR) crisis. Some diagnostic tests that can reliably detect bacterial infections, are primarily available at hospitals and research facilities and not widely accessible in resource-limited settings [14]. At present, no adequate point-of-care (POC) tests exist for distinguishing bacterial from non-bacterial infections in low- and middle-income countries (LMICs).

To address this need, there is an increasing effort at the clinical research- and industry-level to develop POC diagnostic tests that utilize easily measured host biomarkers to discriminate bacterial from non-bacterial infections in patients presenting with AFI. Host biomarkers can be clinical biometric data (e.g., anatomical, physiological, signs and symptoms), biochemical (i.e., inorganic or organic molecules or markers of cellular activity), or genetic markers (i.e., DNA or RNA). Host biomarkers can be detected from any tissue or biological fluid. The goal of a host biomarker assay would be to objectively measure the levels of the biomarker and relate those changes in the biomarker to the indication of disease or biological activity.

Host biomarkers that are currently in clinical use for differentiating bacterial from non-bacterial infections mostly measure nonspecific immunologic responses and inflammation [15]. Dupuy *et*. *al*. (2013) reported that >90% of identified biomarkers have been used in laboratory-based research only, and have not been translated into clinical use [12]. The large body of peer-reviewed literature pertaining to biomarkers therefore represents a unique and largely unexploited repository of host biomarker candidates with potentially more specific discriminatory power in POC formats than markers in current use.

The primary objective of this comprehensive review was to summarize host biomarkers in clinical testing that can discriminate bacterial infections from other types of infection. A review of recently published medical literature was conducted to identify promising host biomarkers and available industry solutions to differentiate bacterial from non-bacterial acute infections.

## Methods

### Inclusion and exclusion criteria

#### Study population

Studies comparing diagnostic performance of host biomarkers in patients with bacterial infections and those with non-bacterial infections were included in this review. The bacterial infection patient groups included either collections of patients with a spectrum of bacterial infections or specific types of bacterial infection (e.g., bacterial meningitis). Non-bacterial infections included viral, fungal, and/or parasitic/protozoan infections. Studies that examined other comparator groups, such as healthy subjects, in addition to the bacterial/non-bacterial infection groups were also included. Studies that only compared host biomarker levels in bacterial infection to non-infectious illnesses, or in sepsis versus non-sepsis illnesses were not included. Studies investigating biomarkers for non-infectious causes were excluded due the large global infectious disease incidence and the critical need for improved case management to reduce AMR.

#### Biomarker types

Studies were restricted to those testing one or more biomarkers produced by the human host. Host biomarkers of primary interest were host proteins, gene transcripts, and biochemical reactions or cellular processes (e.g., erythrocyte sedimentation rate (ESR)). Clinical signs and symptoms (e.g., fever, respiratory rate) were only considered if they were used in combination with host biomarkers or if they were part of an objective (e.g., computerized) fever management algorithm. Host biomarkers that required detection through body imaging procedures were excluded. Any study that examined pathogen markers alone or in combination with host biomarkers was excluded.

#### Study types

Studies that limited their testing to laboratory models, such as animal models or human tissue cultures were excluded. Additionally, studies that were designed to test other research questions using host biomarkers were excluded. Some examples of off-target research questions are those attempting to answer the utility of host biomarkers in: solely diagnosing severity of disease or prognosis of patient, efficacy or safety of vaccines, the impact of host biomarker assay on antibiotic prescribing practices, or cost-effectiveness of biomarker in clinical practice.

#### Time period

This review included the recent literature spanning January 2010 through April 2015. Prior to 2010, many of the published evaluations distinguishing bacterial from non-bacterial infections were dominated by studies on procalcitonin (PCT), C-reactive protein (CRP), and other biomarkers that have since shown variable success as biomarkers of bacterial infection in large clinical trials. Publications in 2010 and later were thus more likely to focus on more recent and newly evaluated biomarkers, and served to filter out biomarkers that were tested earlier and showed low diagnostic performance.

### Data sources

This systematically performed review involved multiple data sources and search strategies. Structured searches were conducted in PubMed, Cochrane Database of Systematic Review (CDSR), and ScienceDaily. Unstructured, free-text web searches (e.g., Google Scholar) were also performed to achieve a highly sensitive search strategy (S1 Table). Free-text searches in PubMed were also conducted separately in order to capture publications ahead of print (e-Pub) and that had not yet been annotated with MeSH terms. Review articles were further assessed for relevant citations of primary studies not captured in the other literature searches.

### Data screening and extraction

All studies identified during the database search were initially manually screened by one author (AK) for their relevance, and abstracts marked for inclusion were further screened by another author (TCR). Full-text articles of all relevant abstracts were retrieved for further review of key information. Citations of the articles in this review were indexed in bibliographic software. The two reviewing authors reached a consensus on the final set of publications that met the review criteria, where adjudication by TCR superseded AK in the event of a tie. Data were recorded in a Microsoft Excel spreadsheet (S1 Appendix), and included article information, author information, study methods, study population, infections/pathogens, clinical information, and biomarker data.

### Quality and validity assessment

Each study selected for full-text review also underwent a quality of evidence review using a modified version of two established quality assessment tools for diagnostic accuracy: Quality Assessment of Diagnostic Accuracy Score (QUADAS) [16] and Lijmer criteria [17, 18]. Each study included in the final review set was assessed against 26 quality factors, each weighted equally (S2 Table). If the quality factor was observed by a study, then a 1-point entry (“Yes”) was made. Any instance where the quality measure was not performed in the study, or it was unclear or not reported by the authors, a zero-point entry (“No”) was entered. All of the “Yes” entries were tallied and a final score was calculated as follows:

(#of "Yes" entries)∕(26criteria×100)=Quality score,%

All studies with a quality measure of >60% were considered “high quality” for the purposes of this analysis.

## Results

### Search results and study characteristics

**Fig 1:**
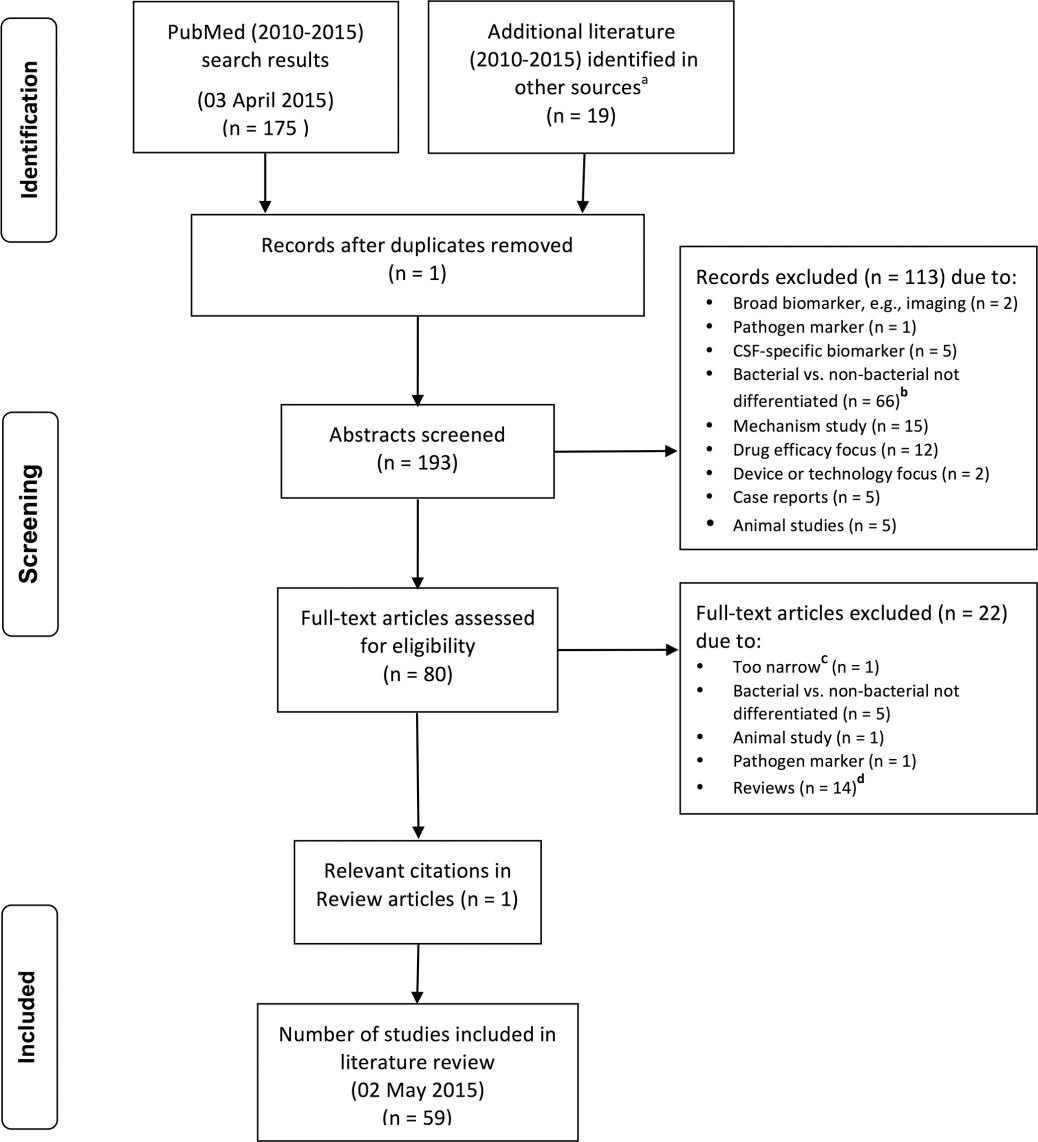
PRISMA flow chart of study selection. (a) Includes Cochrane Database of Systematic Review, Science Daily, and free-text searches online. (b) Includes instances of: detection of a single type of pathogen, measures prognosis/severity, does not differentiate bacterial infection, prevalence studies, and detection of co-infections or cross-reactivity. (c) Study evaluation was limited solely to tick-born infections. (d) Assessed for additional relevant references.

The final set consisted of 59 primary studies published from January 2010 to April 2015 reporting on the diagnostic accuracy of one or more host biomarkers in discriminating bacterial infections from non-bacterial infections (Fig 1).

The 59 publications were conducted in patient populations from over 28 countries, but 5 (8%) systematic reviews and meta-analyses did not report the countries of the primary studies. Additionally, 7 (12%) studies included patients from multiple countries. The majority of studies (49/59; 83%) were conducted in high-income countries (HICs). Only 19% (11/59) of the studies covered populations from LMICs.

**Fig 2:**
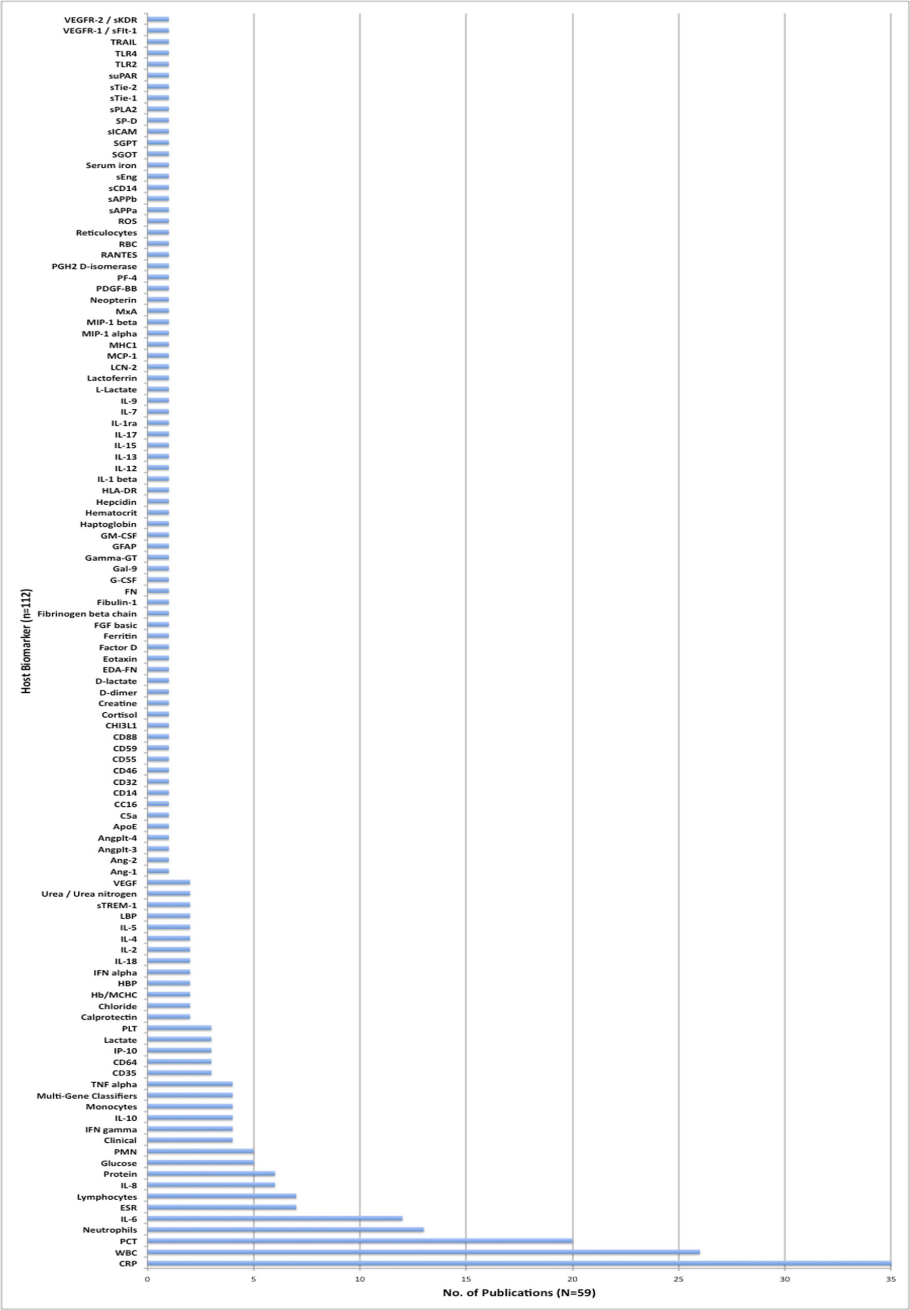
Number of publications that reported on specific biomarkers (2010–2105). The counts in this figure represent the number of publications evaluating a specific host biomarker, regardless of specimen. Biomarker combinations are not represented in this graph. The multi-gene classifier studies screened >1000 host transcripts each, with a final data set of ranging from 10–52 host gene transcripts; however, for the purposes of this graph, a single count was entered for each multi-gene classifier study, regardless of the number of transcripts profiled.

Over 112 unique host biomarkers were reported on in the set of 59 studies. The most frequently evaluated host biomarkers were C-Reactive Protein (CRP, 36; 61%), white blood cell count (WBC, 26; 44%), procalcitonin (PCT, 20; 34%), neutrophil count (absolute/segmented/banded) (13; 22%), and interleukin protein 6 (IL-6,12; 20%). Most biomarkers were only evaluated in a single publication (median 1; range 1–36) (Fig 2).

### Quality assessment

**Fig 3:**
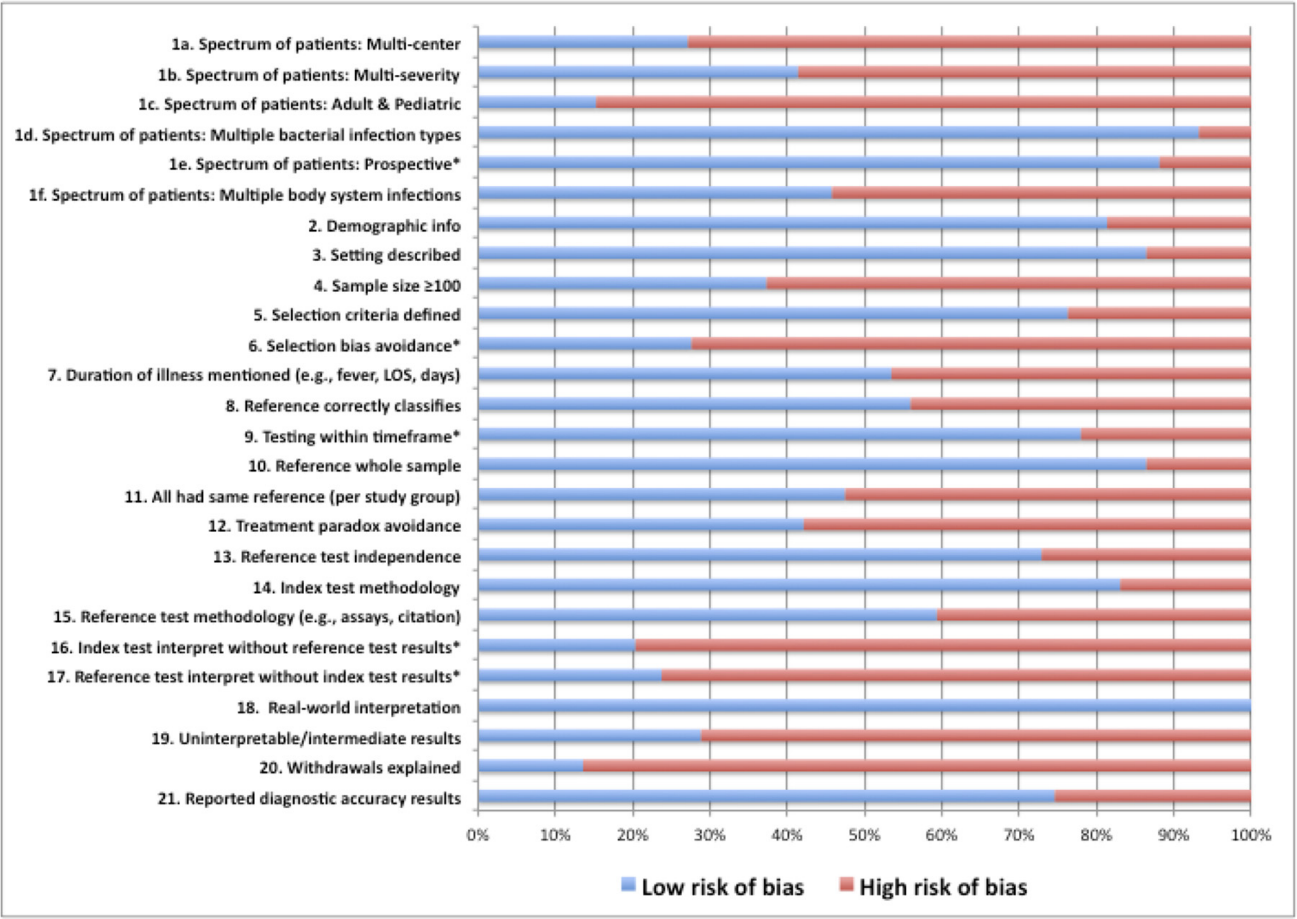
Risk of Bias for 26 Quality Measures: Systematic Review (2010-April 2015). * Criteria that are specified by both QUADAS tool and Lijmer et al. (1999).

Each study was assigned a percentage score derived from the proportion of quality criteria met (S1 Appendix). Among the 59 studies 14 of 26 quality criteria were met by >50% of the studies (Fig 3). Quality scores ranged from 23.1% [19] to 92.3% [20]. The mean score was 56.0% (median 57.7%). In this review, 23 (40.0%) of the studies met the quality score above 60%.

All of the studies included real-world interpretation of study findings and 93.2% of studies evaluated the biomarkers with multiple bacterial infections. However, only 13.6% of studies described or provided the reason for patient withdrawals and only 15.3% of studies included both adult and pediatric populations.

### Diagnostic performance by host biomarker type

#### Blood cells and hematologic markers

The hematologic markers that consistently showed statistically significant differences in bacterial versus non-bacterial infection patients were: polymorphonuclear leukocyte (PMN) counts, neutrophil counts, WBC counts, ESR, red blood cell (RBC) counts, lymphocyte counts, and haptoglobin (Table 1).

**Table 1:** Blood cells and hematologic markers as clinical predictors of bacterial infections ranked by diagnostic performance: comprehensive review 2010–2015.

Biomarker (sample type)	No. Studies	Study Size Range	B vs. NonB^a^	B vs. V^b^	B vs. F^c^	Cut-off Range	% Sensitivity Range (No. studies)	% Specificity Range (No. studies)	Quality Score (%)	No. Ctrs.	Ref.
PMN (blood, CSF)	5 (2 blood, 3 CSF)	28–135	1/1	3/4	0	30 to 49 cells/μl (CSF)	93.3–96 CSF (2)	84.9–95.8 CSF (2)	46.2–65.4	3	**Blood: [30, 32], CSF: [23, 25, 28]**
Neutrophil (blood, CSF, synovial)	13 (10 blood, 2 CNS, 1 synovial)	22–286	1/1	10/11	0/1	4900 to 10000 cells/μl (Blood); 83%, 118 cells/μl (CSF)	42–76 blood (3); 80–90.9 CSF (2)	71.4–88 blood (3); 71.6–85 CSF (2)	30.8–69.2	8	****Blood: [26, 29, 33–40], CSF: [28, 31], synovial [41]****
WBC (blood, CSF, synovial)	28 (1 blood & CSF, 22 blood, 5 CSF, 1 synovial)	22–1743	5/5	16/22	0/1	7200 to 17000 cells/μl (Blood);128 to 10500 cells/μl (CSF)	17–82 blood (6); 66.7–88 CSF (4)	53–82 blood (6); 66–92.5 CSF (4)	30.8–69.2	>29	**Blood:, [22–24, 26, 27, 29, 30, 32–40, 42–48], CSF [21, 23, 25, 28, 31], synovial [41]**
ESR (blood)	7	22–1031	0	5/6	0/1	≥25.5 to ≥32.5 mm/hr	77.4–85 blood	78.3–90 blood	30.8–80.8	8	**[24, 29, 30, 36, 37, 41, 49]**
RBC (CSF)	1	83	0	1/1	0	26 cells/μl (CSF)	57.1 CSF	86.9 CSF	50	1	**[21]**
Monocyte (blood, CSF)	5 (3 blood, 2 CSF)	22–135	0	2/4	0	40% (CSF)	40 CSF (1)	92.4 CSF (1)	46.2–61.5	5	**Blood: [36, 37, 50], CSF: [25, 28]**
Lymphocyte (blood, CSF)	7 (6 blood, 1 CSF)	22–131	0/2	4/6	0/0	-/-	-/-	-/-	30.8–69.2	5	**Blood: [27, 30, 33, 36, 37, 40], CSF: [23]**
Platelet (blood)	3	22–158	0	0/3	0	-/-	-/-	-/-	50–57.7	2	**[36, 38, 39]**
Haptoglobin (CSF)	1	80	0	1/1	0	-/-	-/-	-/-	42.3	1	**[51]**
Hematocrit (blood)	1	22	0	0/1	0	-/-	-/-	-/-	57.7	1	**[36]**
Hemoglobin (blood)	1	22	0	0/1	0	-/-	-/-	-/-	57.7	1	**[36]**
MCHC (blood)	1	22	0	0/1	0	-/-	-/-	-/-	57.7	1	**[36]**
Reticulocyte (blood)	1	22	0	0/1	0	-/-	-/-	-/-	57.7	1	**[36]**

Ctrs, Countries; ESR, Erythrocyte sedimentation rate; MCHC, Mean corpuscular hemoglobin concentration; PMN, Polymorphonuclear leukocyte; RBC, Red blood cell; WBC, White blood cell

a. Bacterial vs. any non-bacterial infection–Statistically significant positive findings out of total number of studies

b. Bacterial vs. any viral infection–Statistically significant positive findings out of total number of studies

c. Bacterial vs. any fungal infection–Statistically significant positive findings out of total number of studies

Note: Columns with footnotes a-c are not mutually exclusive. Entries with “-/-”denote values not reported

WBC and neutrophil counts were the most frequently studied markers (Table 1). The majority of studies (21/28, 75%) investigating WBC counts reported statistically significant differences in patients with bacterial versus non-bacterial infections. Among the 28 studies with WBC counts, only 9 reported diagnostic performance values [21–29]. Studies that evaluated WBC counts in blood reported a high degree of variability in diagnostic performance. All of the studies comparing WBC counts in CSF in patients with bacterial meningitis versus non-bacterial meningitis infections, generally reported high sensitivities and specificities [21, 23, 25, 30, 31] to detect bacterial infections. In CSF, Linder *et al*. (2011) reported the highest combination of sensitivity (88.0%) and specificity (92.5%) at a cut-off of >300 cells/μl [25].

The majority of studies investigating the utility of neutrophil counts (10/13; 77%) reported statistically significant differences in patients with bacterial and non-bacterial infections.

Among the 13 studies with neutrophil counts, only 5 studies reported diagnostic performance values [26, 28, 29, 31, 37]. Two studies comparing neutrophil counts in CSF reported high sensitivities (80%-90.9%), but lower specificities (71.6%-78.3%) for discriminating bacterial from viral meningitis [28, 31]. Neutrophil blood counts provided limited diagnostic value to identify bacterial infections (sensitivity 42%-76%; specificity 71.4%-88%) to distinguish bacterial from non-bacterial infections [26, 29, 37]. Lower blood lymphocyte counts were associated (4/7, 57%) with bacterial infections, although the corresponding diagnostic values were not reported in any of the reviewed studies [30, 33, 37, 40].

The majority (5/7; 71%) of studies that evaluated ESR, a marker for inflammation found it to be a specific marker in differentiating between bacterial and non-bacterial infections [24, 29, 30, 36, 37, 41, 49]. Patients with bacterial infections had a higher ESR than patients with viral infections [30]; [49]; [29, 36, 37]. Two studies reported the diagnostic performance characteristics of ESR, which ranged from 77.4%-85% sensitivity and 78.3%-90% specificity to identify bacterial infections [29].

Out of all investigated hematological markers (Table 1), the highest diagnostic performance to identify bacterial infections was reported for PMN counts (sensitivity: 93–96%; specificity: 85–96%), albeit all investigations were from patients with infections of the central nervous system [25, 28].

#### Inflammation markers

The inflammation markers that showed statistically significant differences in bacterial versus non-bacterial infection patients were: CRP, PCT, calprotectin, soluble angiopoietin 2 receptor (sTie-2), and soluble triggering receptor on myeloid cells (sTREM-1; Table 2). None of the inflammation markers consistently showed high diagnostic performance, although CRP and PCT may have improved diagnostic performance when combined with other biomarkers [20].

**Table 2:** Inflammation markers as clinical predictors of bacterial infections ranked by diagnostic performance: comprehensive review 2010–2015.

Biomarker (sample type)	No. Studies	Study Size Range	B vs. NonB^a^	B vs. V^b^	B vs. F^c^	Cut-off Range	% Sensitivity Range (No. studies)	% Specificity Range (No. studies)	Quality Score (%)	No. Ctrs.	Ref.
CRP (blood)	36	22–1743	6/6	27/28	1/3	>10 to ≥125 mg/L	61.2–100 blood (18)	26–100 blood (18)	30.8–92.3	>20	**[20, 22–24, 26, 28–32, 34–38, 40–42, 44, 46–50, 52–62]**
PCT (blood)	20	22–1743	3/3	15 /16	1/1	0.015 to 1.55 ng/ml	38–97 blood (17)	31–100 blood (17)	30.8–69.2	>17	**[22–24, 26, 31, 33, 39, 40, 43, 46, 53, 55, 56, 58, 60, 62–66]**
Calprotectin (fecal)	2	107–108	0	2/2	0	103.9 to 200 μg/g	87–93 fecal (2)	65–88 fecal (2)	61.5	2	**[48, 49]**
sTie-2 (blood)	1	160	0	1/1	0	≤9.18 ng/ml	72.6 blood (1)	68.1 blood (1)	69.2	1	**[54]**
CC16 (blood)	1	144	0/1	0	0	-/-	-/-	-/-	65.4	1	**[52]**
Neopterin (blood)	1	69	0	0/1	0	-/-	-/-	-/-	30.8	1	**[40]**
sTie-1 (blood)	1	160	0	0/1	0	-/-	-/-	-/-	69.2	1	**[54]**
sTREM-1 (blood)	2	56–300	1/1	1/1	0	755 pg/ml	53.2 blood (1)	86.3 blood (1)	53.8–61.5	2	**[43, 65]**
suPAR (blood)	1	47	0	0/1	0	-/-	-/-	-/-	50.0	1	**[38]**

CC16, Club (Clara) cell protein 16; Ctrs, Countries, CRP, C-reactive protein; PCT, Procalcitonin; sTie-1, Angiopoietin 1 receptor; sTie-2, Angiopoietin 2 receptor; sTREM-1, Soluble triggering receptor on myeloid cells 1; suPAR, Soluble urokinase-type plasminogen activator receptor

a. Bacterial vs. any non-bacterial infection–Statistically significant positive findings out of total number of studies

b. Bacterial vs. any viral infection–Statistically significant positive findings out of total number of studies

c. Bacterial vs. any fungal infection–Statistically significant positive findings out of total number of studies

Note: Columns with footnotes a-c are not mutually exclusive. Entries with “-/-”denote values not reported.

CRP was the most frequently studied host biomarker in this review and the majority of these studies (33/36; 92%) reported statistically significant differences in CRP levels measured in patients with bacterial and those with non-bacterial infections (Table 2) [20, 22–24, 26, 28–32, 34–38, 40–42, 44, 46–50, 52–62]. Half of the studies (18/36; 47%) reported sensitivity and specificity at ranges of 61.2%-100% and 26%-100% respectively to identify bacterial infections [20, 22–24, 26, 28, 29, 31, 34, 37, 40, 42, 48, 50, 53, 56, 58, 60].

All studies comparing CRP levels in blood specimen of patients with meningitis (5/5) reported statistically significant differences in CRP levels between the bacterial and viral group, with higher levels seen in bacterial infections [23, 29–31, 59]. Three studies that included diagnostic performance characteristics for CRP to distinguish between bacterial and viral meningitis found similar sensitivities (73.3%-86%) and a wider range of specificities (78%-94%) to identify bacterial infections [23, 28, 31]. However, all studies also presented data on other host biomarkers that performed with greater diagnostic accuracy than CRP in differentiating bacterial from viral meningitis.

In non-meningitis studies, statistically significant findings for CRP levels were reported in bacterial versus non-bacterial infections, but the related sensitivities and specificities ranged widely across studies. Differences in CRP levels were observed in bacterial versus viral (norovirus) gastroenteritis patients [48], while two studies [41, 53] did not find significant differences in CRP levels. Oguz *et al*. (2011) examined CRP in bacterial and fungal sepsis within a neonatal intensive care population in Turkey and found that CRP levels were lower in bacterial sepsis patients (mean 11.4 mg/l) than in fungal sepsis patients (mean 28.0 mg/l, p = 0.026) [61]. However, they did not report diagnostic performance characteristics.

Ten studies compared CRP levels in bacterial versus viral pneumonia patients [20, 34, 35, 37, 46, 55–58, 62, 65]. A study in Malawian children found significantly higher CRP levels in bacterial infections compared to patients with viral causes (median 185.4 vs. 18.3 mg/l, p<0.001) [56]. However, in children with malaria, CRP levels were not significantly higher during bacterial than viral pneumonia (median 217.4 vs. 96.8 mg/l, p = 0.052), indicating that CRP might be valuable as a diagnostic only after malaria has been ruled out.

PCT was the second most frequently studied host biomarker in this review [22–24, 26, 31, 33, 39, 40, 43, 46, 53, 55, 56, 58, 60, 62–66]. Almost all of the PCT studies (19/20; 95%) reported statistically significant differences in patients with bacterial and non-bacterial infection patients and 17 (85%) studies reported sensitivity and specificity values ranging from 38%-97% and 31%-100%, respectively to identify bacterial infections.

The majority of non-meningitis studies (12/18; 67%) compared PCT levels in bacterial infection patients to those in viral infection patients [24, 26, 33, 39, 40, 55, 56, 58, 60, 63–65]. The Malawian study described earlier found significantly higher PCT levels in bacterial compared to viral infections (median 8.31 ng/ml vs. 0.21 ng/ml, p<0.001) [56]. Again, in children with malaria, PCT levels were not significantly different between bacterial and viral pneumonia infections (median 21.8 vs. 23.1 mg/l, p = 0.825).

While only two studies compared PCT levels in blood specimen of meningitis patients, both found statistically significant higher PCT levels in bacterial meningitis patients [23, 31].

There were 6 other inflammation markers identified in this review, but they were less frequently studied than CRP and PCT (Table 2).

#### Cytokines

In total, 31 cytokine markers were identified in this review (Table 3). The most frequently examined cytokine markers were IL-6 and IL-8. The cytokine markers that showed statistically significant different expression levels in bacterial versus non-bacterial infections were: IL-4, IL-6, IL-8, IL-5, IL-12, IL-13, IL-9, IFN gamma-inducible protein 10 (IP-10; also known as CXC motif chemokine 10, CXC10), platelet factor 4 (PF-4), eotaxin, TNF-related apoptosis-inducing ligand (TRAIL), and granulocyte-macrophage colony-stimulating factor (GM-CSF). A single study reporting diagnostic performance values for IL-4 reported 100% sensitivity, but a specificity of 76.5% [27] to identify bacterial infections. Similarly, IL-8 showed a high diagnostic performance with 82.5%-100% sensitivity and 67.2%-94.0% specificity. IP-10 and TRAIL were of diagnostic value when combined with other biomarkers [20]. Measurements of IL-6, interferon (IFN) gamma, PF-4, and GM-CSF were associated with widely ranging sensitivities and specificities based on individual studies. IL-2, IL-10, and tumor necrosis factor (TNF) alpha showed weak diagnostic performance in distinguishing between bacterial and non-bacterial infections.

**Table 3:** Cytokine markers as clinical predictors of bacterial infections ranked by diagnostic performance: comprehensive review 2010–2015.

Biomarker (sample type)	No. Studies	Study Size Range	B vs. NonB^a^	B vs. V^b^	B vs. F^c^	Cut-off Range	% Sensitivity Range (No. studies)	% Specificity Range (No. studies)	Quality Score (%)	No. Ctrs.	Ref.
**Interleukins**											
IL-4 (blood, saliva)	2 (1 blood & saliva, 1 blood)	76–80	0	2/2	0	9 pg/ml	100 blood (1)	76.5 blood (1)	23.1–57.7	1	**[19, 27]**
IL-6 (blood, CSF, saliva)	12 (1 blood & saliva, 9 blood, 2 CSF)	26–163	2/3	6/8	1/1	0.15 to <74.5 ng/ml (blood), 51.6 ng/ml (CSF)	50–64.3 blood (2); 61.9 CSF (1)	82.8–97.1 blood (2); 95.1 CSF (1)	23.1–80.8	10	**Blood & saliva: [19]**, Blood: **[27, 33, 42, 48–50, 52, 61, 65], CSF: [21, 67]**
IL-8 (blood, CSF, saliva)	6 (1 blood & saliva, 2 blood, 3 CSF)	60–83	0	5/6	0	1.14 to 3600 pg/ml (CSF)	82.5–100 CSF (3)	67.2–94 CSF (3)	23.1–57.7	5	**Blood & saliva: [19]**, Blood: **[27, 33], CSF: [21, 30, 67]**
IL-5 (blood, saliva)	2 (1 blood & saliva, 1 blood)	76–80	0	2/2	0	30 pg/ml (blood)	85.7 blood (1)	67.6 blood (1)	23.1	1	**Blood & saliva: [19, 27]**
IL-10 (blood, saliva)	4 (1 blood & saliva, 3 blood)	76–160	0	0/4	0	-/-	-/-	-/-	23.1–69.2	3	**Blood & saliva: [19, 27, 33, 54]**
IL-18 (blood, saliva)	2	56–160	0	1/2	0	-/-	-/-	-/-	61.5–69.2	1	**[19]**
IL-2 (blood, saliva)	2 (1 blood & saliva, 1 blood)	76–80	0	0/2	0	-/-	-/-	-/-	23.1–57.7	1	**Blood & saliva: [19, 27]**
IL-1 beta (blood, saliva)	1	76	0	0/1	0	-/-	-/-	-/-	23.1	1	**[19]**
IL-12 (blood, saliva)	1	76	0	1/1	0	-/-	-/-	-/-	23.1	1	**[19]**
IL-13 (blood, saliva)	1	76	0	1/1	0	-/-	-/-	-/-	23.1	1	**[19]**
IL-15 (blood, saliva)	1	76	0	0/1	0	-/-	-/-	-/-	23.1	1	**[19]**
IL-17 (blood, saliva)	1	76	0	0/1	0	-/-	-/-	-/-	23.1	1	**[19]**
IL-1ra (blood, saliva)	1	76	0	0/1	0	-/-	-/-	-/-	23.1	1	**[19]**
IL-7 (blood, saliva)	1	76	0	0/1	0	-/-	-/-	-/-	23.1	1	**[19]**
IL-9 (blood, saliva)	1	76	0	1/1	0	-/-	-/-	-/-	23.1	1	**[19]**
**Interferon**											
IFN gamma (blood, saliva)	4 (1 blood & saliva, 3 blood)	76–108	0	2/4	0	0 to 3.42 pg/ml (blood)	40–100 blood (2)	85–88.2 blood (2)	23.1–63.5	3	**Blood & saliva: [19]**, Blood: **[27, 33, 48]**
IFN alpha (blood, saliva)	2 (1 blood & saliva, 1 blood)	76–108	0	1/2	0	-/-	-/-	-/-	23.1–61.5	2	**Blood & saliva: [19]**, Blood: **[48]**
**Chemokine**											
IP-10 (blood, saliva)	3 (1 blood & saliva, 2 blood)	76–765	0	3/3	0	>0.96 ng/ml (blood)	82.3 blood (1)	72.3 blood (1)	23.1–92.3	3	**Blood & saliva: [19]**, Blood: **[20, 54]**
PF-4 (blood)	1	160	0	1/1	0	>29.98 μg/ml	39.8 blood (1)	97.9 blood (1)	69.2	1	**[54]**
Eotaxin (blood, saliva)	1	76	0	1/1	0	-/-	-/-	-/-	23.1	1	**[19]**
MCP-1 (blood, saliva)	1	76	0	0/1	0	-/-	-/-	-/-	23.1	1	**[19]**
MIP-1 alpha (blood, saliva)	1	76	0	0/1	0	-/-	-/-	-/-	23.1	1	**[19]**
MIP-1 beta (blood, saliva)	1	76	0	0/1	0	-/-	-/-	-/-	23.1	1	**[19]**
RANTES (blood, saliva)	1	76	0	0/1	0	-/-	-/-	-/-	23.1	1	**[19]**
**Tumor necrosis factors**											
TNF alpha (blood, saliva)	4 (1 blood & saliva, 3 blood)	76–108	0	0/4	0	-/-	-/-	-/-	23.1–61.5	3	**Blood & saliva: [19]**, Blood: **[27, 33, 48]**
TRAIL (blood)	1	765	0	1/1	0	-/-	-/-	-/-	92.3	1	**[20]**
**Other Cytokines**											
GM (CSF)	1	80	0	1/1	0	30 pg/ml	78.6 CSF (1)	80.9 CSF (1)	57.4	1	**[27]**
VEGF (blood, saliva)	2	76–160	0	1/2	0	-/-	-/-	-/-	23.1–69.2	1	**[19]**
Ang-1 (blood)	1	160	0	0/1	0	-/-	-/-	-/-	69.2	1	**[54]**
Ang-2 (blood)	1	160	0	0/1	0	-/-	-/-	-/-	69.2	1	**[54]**
G-CSF (blood, saliva)	1	76	0	0/1	0	-/-	-/-	-/-	23.1	1	**[19]**

Ang, Angiopoietin; Ctrs, Countries; G-CSF, Granulocyte colony-stimulating factor; GM-CSF, Granulocyte-macrophage colony-stimulating factor; IFN, Interferon; IL, Interleukin; IP-10, IFN gamma-inducible protein 10/CXC motif chemokine 10 (CXCL10); MCP-1, Monocyte chemoattractant protein 1; MIP-1, Macrophage inflammatory protein 1; PF-4, Platelet factor 4; RANTES, Regulated on activation, normal T cell expressed and secreted/Chemokine ligand 5 (CCL5); TNF, Tumor necrosis factor; TRAIL, TNF-related apoptosis-inducing ligand; VEGF, Vascular endothelial growth factor 1/FMS-like tyrosine kinase 1 (Flt1)

a. Bacterial vs. any non-bacterial infection–Statistically significant positive findings out of total number of studies

b. Bacterial vs. any viral infection–Statistically significant positive findings out of total number of studies

c. Bacterial vs. any fungal infection–Statistically significant positive findings out of total number of studies

Note: Columns with footnotes a-c are not mutually exclusive. Entries with “-/-”denote values not reported.

The following cytokine markers were limited to only one study, low quality studies, or did not report diagnostic performance: IL-1 beta, IL-12, IL-13, IL-15, IL-17, IL-1ra, IL-7, IL-9, IL-18, IFN alpha, eotaxin, monocyte chemoattractant protein 1 (MCP-1), macrophage inflammatory protein 1 (MIP-1) alpha, MIP-1 beta, regulated on activation normal T cell expressed and secreted (RANTES; also known as chemokine ligand 5, CCL5), angiopoietin (Ang)-1, Ang-2, granulocyte colony-stimulating factor (G-CSF), and vascular endothelial growth factor 1 (VEGF1; also known as FMS-like tyrosine kinase 1, Flt1). Differences in the levels of interleukin expression in samples from patients with bacterial and non-bacterial infections varied across the interleukin family (Table 3). Sensitivity and specificity data were not frequently reported, but when available, diagnostic performance varied within and across the interleukin family to discriminating bacterial from non-bacterial infections.

The majority of IL-6 (8/12; 67%) and IL-8 studies (5/6; 83.3%) reported statistically significant results to identify bacterial infections. Six other interleukins had at least one study reporting statistically significant differences when comparing their levels in bacterial and non-bacterial infections, with the majority of studies focused on bacterial versus viral infections. IL-4 and IL-5 were evaluated in two studies, both of which reported significant findings [19, 27]. IL-9, IL-12, and IL-13 were each found to be statistically significant in the single study in which they were evaluated [19]. IL-18 was examined in 2 studies, but only one study reported significant findings [54, 65]. For the remaining investigated interleukins no significant differences in their expression levels in patients with bacterial versus non-bacterial infections were found.

Sixteen other cytokines were identified (Table 3). Only half of the studies that evaluated IFN alpha and IFN gamma [19, 27, 33, 48], reported statistically significant results [19, 27, 48]. Among the chemokines, IP-10, PF-4, and eotaxin were each reported as being expressed at significantly different levels in patients with bacterial versus non-bacterial infections [19, 20, 54]. MCP-1, MIP-1 alpha, MIP-1 beta, and RANTES were each evaluated in a single study that reported no significant differences in their levels in saliva or blood of bacterial versus viral upper respiratory tract infections [19].

Two members of the TNF family, TNF alpha and TRAIL, were identified. While TNF alpha was not found to be expressed at significantly different levels in bacterial versus non-bacterial infections in four studies [19, 27, 33, 48], TRAIL was found to be differentially expressed between bacterial and viral infections. However, the study that evaluated TRAIL also determined that diagnostic accuracy is improved if measured with a combination of other inflammation and cytokine markers [20].

Other cytokines identified in this review were GM-CSF [27], VEGF [19, 54], Ang-1 [54], Ang-2 [54], and G-CSF [19] (Table 3). Only GM-CSF and VEGF were associated with statistically significant differences in their expression levels in bacterial versus non-bacterial infection patients [27, 54].

#### Cell surface markers

A total of 13 cell surface biomarkers were identified (Table 4). In general, cell surface markers were infrequently evaluated in discriminating bacterial from non-bacterial infections. The cell surface markers that consistently showed statistically significant differences of expression levels in bacterial versus non-bacterial infections were: cluster of differentiation (CD)64, galectin (Gal)-9, CD35, CD32, major histocompatibility complex class 1 (MHC1), CD88, CD14, CD46, CD55, and CD59. Measurements of Gal-9 were associated with widely ranging sensitivities and specificities. The following cell surface markers were limited to only one study, low quality studies, or did not report diagnostic performance: CD14, CD46, CD59, human leukocyte antigen (HLA)-DR, toll-like receptor (TLR)2, and TLR4. All the cell surface markers from this review were each evaluated in a single study, except for CD35 and CD64, which were each tested in 3 studies. The range of sensitivities and specificities were 65.3%-96% and 68.9%-95.2%, respectively, for the combined group cell surface markers.

**Table 4:** Cell surface markers evaluated as predictors of bacterial infection ranked by diagnostic parameters: comprehensive review 2010–2015.

Biomarker (sample type)	No. Studies	Study Size Range	B vs. NonB^a^	B vs. V^b^	B vs. F^c^	Cut-off Range	% Sensitivity Range (No. studies)	% Specificity Range (No. studies)	Quality Score (%)	No. Ctrs.	Ref.
CD64 (blood)	3	57–1921	2/2	1/1	0	1800 molecules/cell; 1.64 index	71–96 blood (3)	87–95.2 blood (3)	42.3–65.4	3	**[32, 43, 68]**
Gal-9 (blood)	1	63	0	1/1	0	>64.5 pg/ml	81.4 blood (1)	75 blood (1)	30.8	1	**[35]**
CD35 (blood)	3	47–286	0	3/3	0	≥1.5 index	67.5–81 blood (3)	68.9–77 blood (3)	46.2–65.4	2	**[29, 32, 37]**
CD55 (blood)	1	286	0	1/1	0	≥1.25 index	81 blood (1)	77 blood (1)	61.5	1	**[37]**
CD32 (blood)	1	286	0	1/1	0	≥92 to 110,000 molecules/cell	71.4–77.9 blood (1)	72.1–82.0 blood (1)	61.5	1	**[29]**
MHC1 (blood)	1	286	0	1/1	0	≥0.345 ratio	76 blood (1)	91 blood (1)	61.5	1	**[29]**
CD88 (blood)	1	286	0	1/1	0	≥81,804 molecules/cell	65.3 blood (1)	68.9 blood (1)	61.5	1	**[29]**
CD14 (blood)	1	81	0	1/1	0	-/-	-/-	-/-	42.3	1	**[33]**
CD46 (blood)	1	286	0	1/1	0	-/-	-/-	-/-	61.5	1	**[37]**
CD59 (blood)	1	286	0	1/1	0	-/-	-/-	-/-	61.5	1	**[37]**
HLA-DR (blood)	1	81	0	0/1	0	-/-	-/-	-/-	42.3	1	**[33]**
TLR2 (blood)	1	81	0	0/1	0	-/-	-/-	-/-	42.3	1	**[33]**
TLR4 (blood)	1	81	0	0/1	0	-/-	-/-	-/-	42.3	1	**[33]**

CD, Cluster of differentiation; Ctrs, Countries; Gal-9, Galectin 9; HLA-DR, Human leukocyte antigen DR protein complex; MHC1, Major histocompatibility complex class I; TLR, Toll-like receptor

a. Bacterial vs. any non-bacterial infection–Statistically significant positive findings out of total number of studies

b. Bacterial vs. any viral infection–Statistically significant positive findings out of total number of studies

c. Bacterial vs. any fungal infection–Statistically significant positive findings out of total number of studies

Note: Columns with footnotes a-c are not mutually exclusive. Entries with “-/-”denote values not reported.

All studies evaluating CD64 reported statistically significant differences in patients with bacterial versus non-bacterial infections [68], bacterial sepsis versus non-sepsis infections [43], and bacterial versus viral infections [32]. CD64 was also associated with relatively high sensitivity and specificity to identify bacterial infections (Table 4). One study reported that higher diagnostic performance for CD64 in adults (90% sensitivity, 95% sensitivity) compared to pediatrics (71% sensitivity, 87% specificity) when discriminating bacterial from non-bacterial infections [68]. Similarly, all studies evaluating CD35 found statistically significant differences in the CD35 levels in blood specimen from bacteria compared to virus infected patients [29, 32, 37]. However, they also found enhanced diagnostic performance when CD35 was combined with other markers.

Other cell surface biomarkers with statistically significant findings were Gal-9 [35], MHC1 [33], CD14, CD32, CD46, CD55, CD59, and CD88 [29, 37] (Table 4).

#### Metabolic activity markers

There were 13 metabolic activity markers identified in this review (Table 5), with limited number of studies examining each biomarker. The most frequently examined metabolic activity markers were total protein and glucose concentration in CSF. The metabolic activity markers that consistently showed statistically significant different expression levels in bacterial versus non-bacterial infections were: Glucose-CSF, lactate-CSF, protein-CSF, angiopoietin-like protein (Anglpt)-3, reactive oxygen species (ROS), L-lactate-CSF, apolipoprotein E (ApoE), cortisol, urea, and urea nitrogen.

**Table 5:** Metabolic activity markers evaluated as clinical predictors of bacterial infections ranked by diagnostic performance: comprehensive review 2010–2015.

Biomarker (sample type)	No. Studies	Study Size Range	B vs. NonB^a^	B vs. V^b^	B vs. F^c^	Cut-off Range	% Sensitivity Range (No. studies)	% Specificity Range (No. studies)	Quality Score (%)	No. Ctrs.	Ref.
Glucose (CSF)	5	28–253	1/1	3/4	0	2.2 to 2.5 mmol/l; 40 mg/dL	61.1–97 CSF (3)	49–92.3 CSF (3)	50–61.5	5	**[21, 23, 25, 28, 31]**
Lactate (CSF)	3	77–1692	0	3/3	0	3.8 mmol/L	94–96 CSF (2)	94–97 CSF (2)	53.8–61.5	>3	**[25, 31, 69]**
Protein (CSF)	6	28–253	1/1	5/5	0	1000 to 1880 mg/l	84.2–89 CSF (3)	76.9–93.7 CSF (3)	46.2–61.5	5	**[21, 23, 25, 28, 30, 31]**
Angplt-3 (blood)	1	160	0	1/1	0	>135.75 ng/ml	81.4 blood (1)	63.8 blood (1)	69.2	1	**[54]**
ROS (blood)	1	69	0	1/1	0	-/-	75 blood (1)	100 blood (1)	53.8	1	**[70]**
L-Lactate (CSF)	1	83	0	1/1	0	3.3 mmol/l	71.4 CSF (1)	98.4 CSF (1)	50	1	**[21]**
Angplt-4 (blood)	1	160	0	0/1	0	-/-	-/-	-/-	69.2	1	**[54]**
ApoE (CSF)	1	80	0	1/1	0	-/-	-/-	-/-	42.3	1	**[51]**
Cortisol (blood)	1	81	0	1/1	0	-/-	-/-	-/-	42.3	1	**[33]**
Creatine (blood)	1	47	0	0/1	0	-/-	-/-	-/-	50	1	**[38]**
Urea (blood)	1	47	0	1/1	0	-/-	-/-	-/-	50	1	**[38]**
Urea nitrogen (blood)	1	158	0	1/1	0	-/-	-/-	-/-	53.8	1	**[39]**

Angplt, Angiopoietin-like protein; ApoE, Apolipoprotein E; Ctrs, Countries; ROS, Reactive oxygen species

a. Bacterial vs. any non-bacterial infection–Statistically significant positive findings out of total number of studies

b. Bacterial vs. any viral infection–Statistically significant positive findings out of total number of studies

c. Bacterial vs. any fungal infection–Statistically significant positive findings out of total number of studies

Note: Columns with footnotes a-c are not mutually exclusive. Entries with “-/-”denote values not reported.

In CSF, lactate showed high diagnostic performance, but it has not been tested in a broad spectrum of bacterial and non-bacterial infections or as a blood biomarker. ROS, a marker for phagocyte activation, was shown to have 100% diagnostic accuracy in detecting viral infections or 75% accuracy in diagnosing bacterial infections [70]. Measurements of glucose-CSF, protein-CSF, Angplt, and L-lactate-CSF were associated with widely ranging sensitivities and specificities. The following metabolic activity markers were limited to only one study, low quality studies, or no reporting of diagnostic performance: Angplt-4, ApoE, cortisol, creatine, urea, and urea nitrogen.

All six studies that investigated protein in CSF reported statistically significantly higher levels in bacterial versus viral meningitis [21, 23, 25, 28, 30, 31]. Four out of 5 (80%) studies evaluating glucose levels in blood or CSF to discriminate bacterial from viral meningitis reported statistically significant results [21, 23, 25, 28, 31]. Further, lactate levels in the blood or CSF were significantly different in bacterial and viral meningitis patients [25, 31, 69].

Other metabolic activity biomarkers with statistically significant findings were Angplt-3, L-lactate in CSF, ApoE, cortisol, ROS, urea and urea nitrogen (Table 5) [21, 33, 38, 39, 43, 51, 54, 65, 70].

#### Other host biomarkers

In addition to host biomarkers described earlier, 34 miscellaneous host biomarkers were identified (Table 6). As a group, these biomarkers were infrequently examined. Almost all of these biomarkers were only mentioned in one study each, except for chloride-CSF, heparin-binding protein (HBP) and lipopolysaccharide-binding protein (LBP), which were evaluated in two studies each. The biomarkers in this group that consistently showed statistically significant different expression levels in bacterial versus non-bacterial infections were: Chloride-CSF, Serum-iron, myxovirus resistance protein 1 (MxA), LBP, lipocalin (LCN)-2, factor D, lactoferrin, HBP, glial fibrillary acidic protein (GFAP), prostaglandin-H2 (PGH2) D-isomerase, soluble amyloid precursor protein (sAPP)a, sAPPb, secretory phospholipase A2 (sPLA2), D-Lactate-CSF, soluble vascular endothelial growth factor receptor (sVEGFR-2; also known as soluble kinase insert domain receptor, sKDR), soluble intracellular adhesion molecule (sICAM)-1, EDA-containing cellular fibronectin (EDA-FN), soluble endoglin (sEng), fibrinogen beta, fibulin-1, fibronectin (FN), and sCD14. D-lactate in CSF, MxA, and HBP showed high diagnostic performance, but have not been tested in a broad spectrum of bacterial and non-bacterial infections. Measurements of LBP, LCN-2, factor D, lactoferrin, SPLA2, sVEGFR-2, sICAM-1, EDA-FN, and sEng were associated with widely ranging sensitivities and specificities.

**Table 6:** Other host biomarkers evaluated as clinical predictors of bacterial infections ranked by diagnostic performance: comprehensive review 2010–2015.

Biomarker (sample type)	No. Studies	Study Size Range	B vs. NonB^a^	B vs. V^b^	B vs. F^c^	Cut-off Range	% Sensitivity Range (No. studies)	% Specificity Range (No. studies)	Quality Score (%)	No. Ctrs.	Ref.
**Inorganic**											
Chloride (CSF)	2	83–135	0	2/2	0	114 mmol/l;119 mEq/l	52.4–90.9 CSF (2)	80.6–88.5 CSF (2)	50	2	**[21, 28]**
Serum iron (blood)	1	22	0	1/1	0	-/-	-/-	-/-	57.7	1	**[36]**
**Antimicrobial response**											
MxA (blood)	1	60	0	1/1	0	36.7 ng/ml	87.1 blood (1)	90.9 blood (1)	38.5	1	**[44]**
LBP (blood)	2	56–163	1/1	1/1	0	14.6 μg/ml	82 blood (1)	67 blood (1)	61.5	3	**[42, 65]**
LCN-2 (CSF)	1	134	0	1/1	0	-/-	81 CSF (1)	93 CSF (1)	57.7	1	**[59]**
**Complement System**											
Factor D (blood)	1	160	0	1/1 (V vs. B)	0	>1248.1 ng/ml	69 blood (1)	93.6 blood (1)	69.2	1	**[54]**
C5a (blood)	1	160	0	0/1	0	-/-	-/-	-/-	69.2	1	**[54]**
**Homeostasis**											
Lactoferrin (fecal)	1	108	0	1/1	0	97 μg/g	64 fecal (1)	81 fecal (1)	61.5	1	**[48]**
HBP (blood, CSF)	2 (1 blood, 1 CSF)	77–81	0	2/2	0	>20 ng/ml (CSF)	100 CSF (1)	99.2 CSF (1)	42.3–61.5	2	**Blood: [33], CSF: [25]**
Ferritin (blood)	1	22	0	0/1	0	-/-	-/-	-/-	57.7	1	**[36]**
Hepcidin (blood)	1	22	0	0/1	0	-/-	-/-	-/-	57.7	1	**[36]**
**Neuronal**											
GFAP (CSF)	1	80	0	1/1	0	-/-	-/-	-/-	42.3	1	**[51]**
PGH2 D-isomerase (CSF)	1	80	0	1/1	0	-/-	-/-	-/-	42.3	1	**[51]**
sAPPa (CSF)	1	80	0	1/1	0	-/-	-/-	-/-	42.3	1	**[51]**
sAPPb (CSF)	1	80	0	1/1	0	-/-	-/-	-/-	42.3	1	**[51]**
**Other**											
sPLA2 (blood)	1	76	0	1/1	0	20 to 100 ng/ml	64–93 blood (1)	67–98 blood (1)	65.4	1	**[45]**
D-Lactate (CSF)	1	83	0	1/1	0	12.8 μmol/l	94.7 CSF (1)	79.7 CSF (1)	50	1	**[21]**
Soluble VEGFR-2 (blood)	1	160	0	1/1	0	>5.18 ng/ml	84.1 blood (1)	51.1 blood (1)	69.2	1	**[54]**
Soluble ICAM-1 (blood)	1	160	0	1/1	0	>285.9 ng/ml	83.2 blood (1)	78.8 blood (1)	69.2	1	**[54]**
EDA-FN (blood, CSF)	1 (blood & CSF)	85	0	1/1	0	-/-	83 (blood & CSF (1)	89 blood & CSF (1)	46.2	1	**[71]**
sEng (blood)	1	160	0	1/1	0	>9.12 ng/ml	79.7 blood (1)	93.6 blood (1)	69.2	1	**[54]**
CHI3L1 (blood)	1	160	0	0/1	0	-/-	-/-	-/-	69.2	1	**[54]**
D-dimer (blood)	1	144	0/1	0	0	-/-	-/-	-/-	65.4	1	**[52]**
FGF (blood, saliva)	1	76	0	0/1	0	-/-	-/-	-/-	23.1	1	**[19]**
Fibrinogen beta (CSF)	1	80	0	1/1	0	-/-	-/-	-/-	42.3	1	**[51]**
Fibulin-1 (CSF)	1	80	0	1/1	0	-/-	-/-	-/-	42.3	1	**[51]**
FN (blood, CSF)	1	85	0	1/1	0	-/-	-/-	-/-	46.2	1	**[71]**
Gamma-GT (blood)	1	47	0	0/1	0	-/-	-/-	-/-	50	1	**[38]**
PDGF-BB (blood, saliva)	1	76	0	0/1	0	-/-	-/-	-/-	23.1	1	**[19]**
Soluble CD14 (blood)	1	81	0	1/1	0	-/-	-/-	-/-	42.3	1	**[33]**
SGOT (blood)	1	47	0	0 /1	0	-/-	-/-	-/-	50	1	**[38]**
SGPT (blood)	1	47	0	0/1	0	-/-	-/-	-/-	50	1	**[38]**
SP-D (blood)	1	144	0/1	0	0	-/-	-/-	-/-	65.4	1	**[52]**
Soluble VEGFR-1 (blood)	1	160	0	0/1	0	-/-	-/-	-/-	69.2	1	**[54]**

C5a, Complement component 5a; CHI3L1, Chitinase 3-like protein 1; Ctrs, Countries; EDA-FN, EDA-containing cellular fibronectin; FGF, Fibroblast growth factor; FN, Fibronectin; Gamma-GT, Gamma-glutamyl transpeptidase; GFAP, Glial fibrillary acidic protein; HBP, Heparin-binding protein; LBP, Lipopolysaccharide-binding protein; LCN-2, Lipocalin-2; MxA, Myxovirus resistance protein 1; PDGF-BB, Platelet-derived growth factor homodimer BB; PGH2 D-isomerase, Prostaglandin-H2 D-isomerase; sAPP, Soluble amyloid precursor protein; sCD14, Soluble cluster of differentiation protein 14; sEng, Soluble endoglin; SGOT, Serum glutamic-oxaloacetic transaminase; SGPT, Serum glutamic-pyruvic transaminase; sICAM-1, Soluble intracellular adhesion molecule-1/Soluble CD54; SP-D, Surfactant protein D; sPLA2, Secretory phospholipase A2; VEGFR-1, Vascular endothelial growth factor receptor 1/soluble Fms-like tyrosine kinase 1 (sFlt1); VEGFR-2, Vascular endothelial growth factor receptor 2/soluble kinase insert domain receptor (sKDR)

a. Bacterial vs. any non-bacterial infection–Statistically significant positive findings out of total number of studies

b. Bacterial vs. any viral infection–Statistically significant positive findings out of total number of studies

c. Bacterial vs. any fungal infection–Statistically significant positive findings out of total number of studies

Note: Columns with footnotes a-c are not mutually exclusive. Entries with “-/-”denote values not reported.

A large number of biomarkers in this group were limited to only one study (Table 6).

Of the studies that evaluated inorganic molecules, chloride in CSF [21, 28] and serum iron [36] reported statistically significant differences in the levels observed in bacterial versus non-bacterial infection patients. Chloride concentrations in CSF were compared in two meningitis studies, but the sensitivities differed greatly. No diagnostic performance measures were reported for the serum iron evaluation.

Statistically significant results of HBP in blood samples from bacterial versus viral infections [33] or in CSF samples from bacterial versus viral meningitis patients [25] were found. The diagnostic performance of HBP is one of the highest diagnostic values identified in this review. In addition, Kawamura *et al*. (2012) reported a relatively high sensitivity (87.1%) and specificity (90.9%) to identify viral infections for MxA in their study of pediatric cases of bacterial (n = 11) and viral infection (n = 11) in Japan [44].

Out of the 34 miscellaneous biomarkers, 12 biomarkers–c5a, ferritin, hepcidin, CHI3L1, D-dimer, FGF, Gamma-GT, PDGF-BB, SGOT, SGPT, SP-D, and VEGFR-1– did not have any statistically significant data to support their use in differentiating bacterial versus non-bacterial infections (Table 6) [19, 36, 38, 52, 54]. However, these biomarkers were examined only within one study each with quality scores ranging from 23.1% to 69.2%. Other biomarkers with one to two studies showing statistically significant differences in expression levels in bacterial infection patients compared to non-bacterial infection patients include LBP, LCN-2, lactoferrin, sPLA2, D-Lactate in CSF, EDA-FN, FN, and soluble CD14. FN and sCD14 did not have any sensitivity or specificity data reported in this review.

#### Host transcription signatures

Four host transcriptional profiling studies were identified [39, 72–74] that each screened several thousand transcripts and eventually described 10 to 52 host transcripts that accurately classified the type of infection (Table 7). All of these studies used blood as the sample of choice. Two studies involved US patients, one included Scottish patients, and a fourth study had a multinational (Australian, UK, and US) study population.

**Table 7:** Summary of multi-gene classifiers: comprehensive review 2010–2015.

Reference	Comparison / Performance	Multi-gene classifier		
Hu (2013)[72]	Acute bacterial vs. Febrile viral infection / Not specified	*ACTR2*	*IFNGR2*	***OAS2***
		*AGER*	***ISG15***	***OAS3***
		*ARAP3*	*ITGA2B*	***OASL***
		*EP300*	***ITGAM***	*OSBPL8*
		*F13A1*	*ITGAX*	*OTOF*
		*GNG11*	*ITGB3*	*PROS1*
		***HERC5***	*ITGB5*	***RSAD2***
		***IFI27***	*MAP2K4*	*SORL1*
		***IFI6***	*MT2A*	*SPATS2L*
		***IFIT1***	*MYL9*	*VHL*
		*IFNGR1*	***OAS1***	*ZYX*
Smith (2014)[73]	Bacterial sepsis vs. Non bacterial or Healthy / Sensitivity: 100%, Specificity: 100%	*ALPL*	*GYG1*	*MPO*
		*C19orf59*	*HK3*	*ORM1*
		*CD247*	*HLA-DMB*	*PFKFB3*
		*CD3D*	*HP*	*PGYRP1*
		*CD7*	*IFITM3*	*PRTN3*
		*CEACAM1*	IL18R1*	*PSTPI2*
		*CKAP4*	IL18R1*	*RETN*
		*CSF3R*	*IL1R2*	*RNF24*
		*DYSF*	*IL1RN*	*S100A12*
		*FCGR1A*	***ITGAM***	*SLC2A3*
		*FFAR2*	*ITM2A*	*SP11*
		FGR*	*LCN2*	*SRCAP-like*
		*FPR2*	*LIME1*	*STXBP2*
		*FPR84*	*LRRN3*	***TNFAIP6***
		*G4GALT5*	*MAL*	*TRAJ17*
		*GRAP*	*MMP9*	*TRBV28*
		*GRINA*		
Suarez (2015)[39]	Bacterial vs. Viral lower respiratory tract infection / Sensitivity: 38%, Specificity: 91%	*BTN3A3*	*IFIT3*	***OASL***
		***IFI27***	*KIAA1618*	*PARP9*
		***IFI44***	***OAS2***	***RSAD2***
		***IFIT2***		
Zaas (2013)[74]	Viral influenza vs. Bacterial respiratory infections / Sensitivity: 89%, Specificity: 94%	*ADAR*	***IFIT1***	***OAS2***
		*ATF3*	***IFIT2***	***OAS3***
		*C13orf18*	***IFIT3***	***OASL***
		*CCL2*	*IFIT5*	*PPIA*
		*CTSL1*	*IL16*	*PRSS21*
		*CUZD1*	***ISG15***	*RPL30*
		*DDX58*	*LAMP3*	***RSAD2***
		*ENOSF1*	*LILRB2*	*RTP4*
		*GAPDH*	*LILRB1*	*4-Sep*
		*GBP1*	*LOC26010*	*SERPING1*
		*GM2A*	*LY6E*	*SIGLEC1*
		***HERC5***	*MX1*	*SOCS1*
		*HLA-DOB*	*NDUFA10*	*SOCS2*
		***IFI27***	*NLRP3*	*SOCS5*
		***IFI44***	*NOD2*	***TNFAIP6***
		*IFI44L*	***OAS1***	*XAF1*
		***IFI6***		

* FGR and IL18R1 were each listed twice in the final gene set in Smith et al. (2014) without further explanation.

Entries in bold font appear in more than one host transcriptional profiling study in this review

The study by Hu *et al*. (2013) found that cytosolic pattern recognition receptors, which activate IFN regulatory factors, were up-regulated in febrile viral patients while genes in the integrin signaling pathway were activated only in bacterial infections [72]. They also reported 88%-91% accuracy using a 33-gene classifier.

Smith *et al*. (2014) identified a 52-gene classifier consisting of the following functional pathways: innate immunity, adaptive immunity, and sugar and lipid metabolic pathways [73]. In the replication and validation testing, the 52-gene classifier set performed with 100% sensitivity and 100% specificity in discriminating bacterial sepsis in neonates from healthy control patients.

Further, Suarez *et al*. (2015) identified a 10-gene classifier for discriminating bacterial from viral lower respiratory tract infection [39]. Eight of ten classifiers were interferon-related genes (*IFI44*, *IFIT3*, *IFI27*, *RSAD2*, *OAS2*, *OASL*, *IFIT2*, *and PARP9*). They also measured WBC counts, neutrophil counts, platelet counts, PCT levels, and serum urea nitrogen concentration. They determined that a combination of the 10-gene classifier with PCT provided the greatest diagnostic accuracy in discriminating bacterial from viral lower respiratory tract infections.

Further, Zaas *et al*. (2013) identified a 48-gene classifier that had 100% accuracy for detection of H3N2-influenza and 87% accuracy for detection of H1N1-influenza [74]. They also tested the 48-gene classifier in the emergency room patients and determined 89% sensitivity and 94% specificity in discriminating viral respiratory infections from bacterial infections.

#### Combination of host biomarkers

There were five studies that reported performance measures of biomarker combinations [20, 29, 32, 37, 43]. Three studies compared blood cell counts and blood cell surface markers individually and in combination for the purposes of discriminating bacterial from non-bacterial infections [29, 32, 37]. In each study, the cell surface markers that showed the greatest differences in expression levels in blood samples taken from bacterial and non-bacterial infection patients, were then tested for enhanced performance as a combination marker. Mokuda *et al*. (2012) observed a lower sensitivity (67%), but higher specificity (80%) to identify bacterial infections by evaluating the combined expression of CD34/CD64, rather than using a single marker [32]. Nuutila *et al*. (2013) conducted two separate studies that examined a combination of host cell surface biomarkers. Together, CD35 + CD55 were 81% sensitive and 77% specific [37], while the combination of four cell surface markers, CD35 + CD32 + CD88 + MHC1, resulted in a sensitivity of 90.9% and specificity of 91.8% [29].

Other studies examined unrelated biomolecules in combination. For example, Gibot *et*. *al*. (2012) combined WBC count, PCT, sTREM-1, and CD64 on neutrophils in discriminating bacterial sepsis (n = 146) from non-sepsis cases (n = 154) [43]. Each of the biomarkers was an independent predictor of infection, with the best sensitivity/specificity values in the CD64 biomarker. However, the combination of PCT, sTREM-1, and CD64 performed significantly better than each of the biomarkers evaluated individually (p<0.001). The combination of biomarkers was able to identify >90% of the sepsis patients in the validation cohort.

Another example of enhanced diagnostic performance in combination of pathway-unrelated biomarkers is the study by Oved *et al*. (2015) [20]. They found that CRP, TRAIL, and IP-10 were each independent biomarkers for discriminating bacterial from viral infections. Additionally, CRP expression was induced in bacterial infections, whereas TRAIL and IP-10 expression were induced during viral infections (as compared to non-infectious controls). The combination of bacterially-induced and virally-induced biomarkers was a more robust method (p<0.001) for discriminating bacterial from viral infection than CRP, TRAIL, or IP-10 individually, as well as other routinely used clinical parameters and other combinations of biomarkers (p<0.001). The 3-marker combination of CRP + TRAIL + IP-10 was 95% sensitive and 91% specific in the microbiologically-confirmed subgroup.

A single study by Suarez *et al*. (2015) examined the combination of host genetic and non-genetic biomarkers in a US adult population with lower respiratory tract infections [39]. The authors were able to identify a 10-gene classifier for discriminating bacterial from viral lower respiratory tract infection (Table 7). They also measured WBC counts, neutrophil counts, platelet counts, PCT levels, and serum urea nitrogen concentration and the combination of the 10-gene signature plus PCT provided the greatest diagnostic accuracy in discriminating bacterial from viral lower respiratory tract infections, with a sensitivity of 95% (vs. 38% for PCT alone) and specificity of 92% (vs. 91% for PCT alone).

This review did not systematically evaluate the value of clinical assessment methods, however three studies were identified that focused on quantitative, primarily objective clinical algorithms (e.g., utilizing computerized scoring or applying standardized data capture) that have been used for discriminating bacterial from non-bacterial infections [11, 75, 76]. None of the studies in this review evaluated host biomarkers in combination with clinical assessments; however, Brodska *et al*. (2013) mentioned that PCT in conjunction with clinical biometric data might improve the discrimination between bacterial versus fungal sepsis [53].

### Summary of host biomarkers with high diagnostic performance

Table 8 shows the host biomarkers and combinations of biomarkers that had a sensitivity and specificity ≥85% for identification of bacterial infections, or had a sensitivity or specificity of at least 100% with the other (sensitivity or specificity) >75. Based on the available data we determined that these biomarkers and combinations of biomarkers had the greatest potential for future clinical utility, but also that all would need comprehensive performance evaluations in well-planned clinical studies, particularly in low- and middle-income countries.

**Table 8:** Summary of high-performing host biomarkers with statistically significant findings.

Biomarker	No. Significant Studies	Study Quality Score (%)	Infections (Specimen)	Patients (No. studies)	Sensitivity (%)	Specificity (%)	Cut-off	Ref.
HBP	2	42.3–61.5	B/V meningitis (CSF), B/V (Blood)	Adults (2)	100 (CSF)	99.2 (CSF)	>20 ng/ml	**[25, 33]**
CRP +IP-10 +TRAIL	1	92.3	B/V (Blood)	Adults + Pediatrics (1)	95 (Blood)	91 (Blood)	CRP B ~135 vs. V ~125 μg/ml; IP-10 B ~600 vs. V ~800 pg/ml, TRAIL B ~50 vs. V ~150 pg/ml	**[20]**
Lactate	3	53.8–61.5	B/V meningitis/ encephalitis (CSF)	Adults (2), Adults + Pediatrics (1)	94–96 (CSF)	94–97 (CSF)	3.8 mmol/l	**[25, 31, 69]**
PCT +10-Gene classifier	1	53.8	B/V lower respiratory tract (Blood)	Adults (1)	95 (Blood)	92 (Blood)	N/A	**[39]**
PMN counts	4 (5 total)	46.2–65.4	B/V meningitis (CSF, Blood), B/V (Blood)	Adults (4), Pediatrics (1)	93.3–96 (CSF)	84.9–95.8 (CSF)	30–49 cells/μl	**[23, 25, 30, 32]**
48-Gene classifier	1	84.6	V/B respiratory infection (Blood)	Adults (1)	89 (Blood)	94 (Blood)	N/A	**[74]**
CD35 +CD32 +CD88 +MHC1	1	61.5	B/V (Blood)	Adults (1)	90.9 (Blood)	91.8 (Blood)	CD35 B 151x10^3^ vs. V 45x10^3^ cells/neutrophil; CD32 B 158x10 vs. V 65x10^3^ cells/monocyte; CD88 B 112x10^3^ vs. V 47x10^3^ cells/monocyte; MHC1 B 0.40 vs. V 0.28 ratio	**[29]**
MxA	1	38.5	V/B (Blood)	Pediatrics (1)	87.1 (Blood)	90.9 (Blood)	36.7 ng/ml	**[44]**
IL-4	2	23.1–57.7	B/V upper respiratory tract (Blood, Saliva), B pneumonia/V influenza (Blood)	Adults (2)	100 (Blood)	76.5 (Blood)	9 pg/ml	**[19, 27]**

B, Bacterial; CD, Cluster of differentiation; CRP, C-reactive protein; HBP, Heparin-binding protein; IL, interleukin; IP-10, IFN gamma-inducible protein 10/CXC motif chemokine 10 (CXCL10); MHC, Major histocompatibility complex; MxA, Myxovirus resistance protein 1; N/A, Not applicable; NR, Not reported; PCT, Procalcitonin; PMN, Polymorphonuclear leukocyte; TRAIL, TNF-related apoptosis-inducing ligand; V, Viral.

### Commercialized biomarkers for point-of-care

Within the study period only, the results of the Immuno*X*pert^TM^ test which is CE-IVD certified for commercial use in select countries, were published. This assay detects levels of CRP + IP-10 + TRAIL and the combination of biomarkers showed high sensitivity and specificity for identifying bacterial and viral infections (sensitivity 95%; specificity 91%). This product (currently ELISA based) has near-term potential as POC diagnostics but the clinical trials of these devices so far have been limited to studies in HICs

## Discussion

In total, 59 articles published from 2010 through April 2015 assessed the diagnostic performance of over 112 unique host biomarkers to discriminating bacterial from non-bacterial infections. The most frequently evaluated host biomarkers identified in publications from the past five years were CRP, WBC, PCT, neutrophil count, and IL-6. One of the best performing host biomarkers identified was HBP, albeit only in two studies [25]. Several of the high performing biomarkers were combinations of host biomarkers or combinations including protein biomarkers and gene-classifiers. While many of the identified host biomarkers are currently available in commercial assays (i.e. blood cell counts, ESR, CRP, PCT, calprotectin), most existing assays are not specifically designed to differentiate bacterial from non-bacterial infections. Further, none of the evaluated biomarkers are currently available as simple POC tests suitable for deployment at the lowest level of the health care system. It should be noted that two promising studies, using commercially available POC biomarker tests, were identified after the study inclusion cut-off (April 2015). The published performance evaluation data from the two assays appears to be promising. FebriDx^TM^ (based on POC detection of MxA+CRP), demonstrated 80% sensitivity and 92% specificity for detecting bacterial causes of fever, and SeptiCyte® (a proprietary gene-classifier) demonstrated an AUC of 0.92 in discrimination of infectious sepsis cases from non-infectious controls [77, 78]. Like most other host biomarker assays, these were unfortunately only studied in hospital settings in HICs and require additional clinical evaluation, particularly in LMICs.

This review identified a large body of literature on host biomarkers evaluated in HICs. The study populations were most frequently from USA, Japan, Germany, and France, whereas less than one-fifth of the studies included populations from LMICs. With an estimated 600 million cases of acute fever recorded in African children in 2007 [79], the need for a fever triage assay for these environments is great. Febrile illnesses in these regions are often managed with antibiotic treatment without confirmation of the causative agent due to a lack of rapid diagnostic tests, leading to a rise in AMR. However, as awareness of this need grows, an increasing number of studies describing the utility of host biomarkers for bacterial infection diagnosis in LMICs are being published [80–85].

Despite the emergence of novel biomarkers, biomarker combinations and biomarker detection strategies in the past five years, there continue to be many knowledge gaps. Most of the novel biomarkers with strong performance values identified in this review were only evaluated in a small number of patients, and the combined study quality scores ranged widely from 23.1% to 92.3%, indicating that there is a need for standardizing biomarker study methods and reporting performance results. There were also several host biomarkers that were repeatedly shown not to be effective markers for discriminating between bacterial and non-bacterial infections (i.e. RBC counts, platelet counts, IL-10, IL-2, and TNF alpha) and unless there is a compelling reason to continue to pursue these markers it seems future efforts might best be spent on other targets. Further, evaluations of a number of biomarkers (i.e. ApoE, IFN alpha, FGF, ferritin) did not include appropriate statistical measures and their diagnostic potential needs to be considered undetermined at this time. Additionally, more clinical studies are needed to explore the utility of the host biomarkers in different age groups.

Blood, saliva, nasal swabs, and sputum are more accessible specimen types than CSF for POC diagnostics and studies that relied solely on evaluations within CSF should be repeated in other specimen types. An additional consideration is the practicality of implementing gene-classifier systems as POC tests at different settings of the health care system. The current review also indicated that over three-fourths of biomarker evaluation studies did not: include populations from multiple sites or LMICs; recruit patients consecutively; explain patient withdrawals from studies; include both adult and pediatric patients; interpret the biomarker results in a blinded manner; or interpret the reference test without knowledge of the biomarker test results. These are all design issues that can lead to study bias and limit the quality and generalizability of the findings and need to be addressed in future work. An additional major factor in the wide variety of trial methods and reported results is that reference standards for determining whether a patient is infected primarily by a bacterial or non-bacterial infection is poorly defined. Likewise the interactions between microbiome communities and biomarkers are poorly understood and basic research needs to address these issues further.

### Limitations

While there are many strengths to this comprehensive review, some limitations should also be recognized. This review was intentionally designed to evaluate only the recent literature spanning 2010 through April 2015 as the literature prior to this was dominated by evaluations of the discriminatory utility of PCT and CRP and other biomarkers that had failed to provide sufficient discriminatory power in rigorous clinical trials. The rationale was to focus on the more recent literature to determine if recently discovered biomarkers had better performance than most of the previously identified candidates. However, it is possible that the use of the narrow time period excluded other promising candidates before or after the inclusion period and limited the amount of data obtained for well-established biomarkers, such as CRP and PCT.

This review had a very specific scope of interest, which was to identify host biomarkers that were evaluated for diagnostic performance in discriminating bacterial from non-bacterial infections in clinical trials. Laboratory studies that examined host biomarker performance for detecting bacterial infection without the associated data on non-bacterial infection patients were excluded from this review. It is possible that additional viable host biomarker candidates were excluded due to this specific search criterion.

The quality assessment method we used utilized multiple published criteria for evaluating diagnostic studies to improve objectivity. If information to assess particular quality criteria were not reported or were not described sufficiently/clearly to understand, then for the purposes of this review, it was assumed that those criteria were not met by the study. It is possible that poor quality of reporting was assessed in this review as poor quality of study method.

### Future outlook

The results of this review can be used to help guide future research in this arena and help identify the most promising marker for future use. The combination of laboratory-based biomarker testing in combination with clinical algorithms has shown great promise in preliminary studies in Africa and by improving the biomarker component, patient outcome might be further improved [86].

The gaps outlined above need to be addressed, ideally collaboratively, by industry, academia, international health organizations and other institutions with the aligned goal of 1) identifying promising host biomarkers that can distinguish bacterial from non-bacterial infections, 2) developing these promising biomarkers into affordable rapid POC tests with practical implementation and utility in LMICs, 3) establishing standardized quality criteria for testing and development, and 4) commercializing the tests after thorough validation in clinical settings.

## Supporting Information

S1 PRISMA Checklist (DOC)

S1 Appendix Literature data extraction. (XLSX)

S1 Table Electronic search strategies. (DOCX)

S2 Table List of 26 quality criteria used to assess bias, generalizability, and validity of diagnostic accuracy and performance studies. (DOCX)
