# Supplementary material for: Host Biomarkers for Distinguishing Bacterial from Non-Bacterial Causes of Acute Febrile Illness: A Comprehensive Review
Source: PLoS One. 2016 Aug 3;11(8):e0160278. doi: 10.1371/journal.pone.0160278 (PMC4972355; doi:10.1371/journal.pone.0160278)
Supplement: S1 Table — (DOCX) [file pone.0160278.s003.docx]

| **Data Source** | **Criteria** | **Actual Search Terms** |
| --- | --- | --- |
| PubMed <http://www.pubmed.gov> | Biomarkers | "biological markers"[Mesh] NOT "Tumor Markers, Biological"[Mesh] |
|  | AND Bacterial infections | AND ("Bacterial Infections/blood"[Mesh] OR "Bacterial Infections/cerebrospinal fluid"[Mesh] OR "Bacterial Infections/diagnosis"[Mesh] OR "Bacterial Infections/urine"[Mesh] OR "Sepsis/blood"[Mesh] OR "Sepsis/cerebrospinal fluid"[Mesh] OR "Sepsis/diagnosis"[Mesh] OR "Sepsis/urine"[Mesh] OR ("Bacterial Infections/diagnosis"[Mesh] AND ("Bodily Secretions/diagnostic use"[Mesh] OR "Bodily Secretions/microbiology"[Mesh] OR "Oropharynx/analysis"[Mesh] OR "Oropharynx/microbiology"[Mesh] OR "Oropharynx/secretion"[Mesh] OR "Nasopharynx/analysis"[Mesh] OR "Nasopharynx/microbiology"[Mesh] OR "Nasopharynx/secretion"[Mesh] OR "cerebrospinal fluid"[Subheading] OR "Cerebrospinal Fluid/analysis"[Mesh] OR "Cerebrospinal Fluid/microbiology"[Mesh] OR "Cerebrospinal Fluid/secretion"[Mesh] OR "urine"[Subheading] OR "Urine/analysis"[Mesh] OR "Urine/microbiology"[Mesh] OR "Urine/secretion"[Mesh] OR "blood"[Subheading] OR "Blood/analysis"[Mesh] OR "Blood/diagnostic use"[Mesh] OR "Blood/microbiology"[Mesh] OR "Blood/secretion"[Mesh] OR "Saliva/analysis"[Mesh] OR "Saliva/microbiology"[Mesh] OR "Saliva/secretion"[Mesh] OR "nasal swab"[All Fields])) |
|  | AND Viral/ Fungal/ Parasitic/Protozoan Infections | AND ("Virus Diseases/blood"[Mesh] OR "Virus Diseases/cerebrospinal fluid"[Mesh] OR "Virus Diseases/diagnosis"[Mesh] OR "Virus Diseases/urine"[Mesh] OR "Malaria/blood"[Mesh] OR "Malaria/cerebrospinal fluid"[Mesh] OR "Malaria/diagnosis"[Mesh] OR "Malaria/urine"[Mesh] OR "Mycoses/blood"[Mesh] OR "Mycoses/cerebrospinal fluid"[Mesh] OR "Mycoses/diagnosis"[Mesh] OR "Mycoses/urine"[Mesh] OR "Protozoan Infections/blood"[Mesh] OR "Protozoan Infections/cerebrospinal fluid"[Mesh] OR "Protozoan Infections/diagnosis"[Mesh] OR "Protozoan Infections/urine"[Mesh] OR "Blood/parasitology"[Mesh] OR ("Virus Diseases/diagnosis"[Mesh] AND ("Bodily Secretions/diagnostic use"[Mesh] OR "Bodily Secretions/virology"[Mesh] OR "Oropharynx/analysis"[Mesh] OR "Oropharynx/secretion"[Mesh] OR "Oropharynx/virology"[Mesh] OR "Nasopharynx/analysis"[Mesh] OR "Nasopharynx/secretion"[Mesh] OR "Nasopharynx/virology"[Mesh] OR "cerebrospinal fluid"[Subheading] OR "Cerebrospinal Fluid/analysis"[Mesh] OR "Cerebrospinal Fluid/secretion"[Mesh] OR "Cerebrospinal Fluid/virology"[Mesh] OR "urine"[Subheading] OR "Urine/analysis"[Mesh] OR "Urine/secretion"[Mesh] OR "Urine/virology"[Mesh] OR "blood"[Subheading] OR "Blood/analysis"[Mesh] OR "Blood/diagnostic use"[Mesh] OR "Blood/secretion"[Mesh] OR "Blood/virology"[Mesh] OR "Saliva/analysis"[Mesh] OR "Saliva/secretion"[Mesh] OR "Saliva/virology"[Mesh] OR "nasal swab"[All Fields])) |
|  | NOT Vaccines | NOT "Vaccines"[Mesh] |
|  | Limit to human populations | AND "humans"[MeSH Terms] |
|  | Limit to English language articles | AND English[lang] |
|  | Limit to publications in last 5 years | AND "2010/01/01"[PDAT]: "2015/12/31"[PDAT] |
| Cochrane Database of Systematic Reviews (CDSR) <http://www.thecochranelibrary.com> | Bacterial infections | “bacter*” |
|  | AND Viral/ Fungal/ Parasitic/Protozoan Infections | AND (“vir*” OR “fung*” OR “parasit*”) |
| ScienceDaily <http://www.sciencedaily.com> | Bacterial/ Viral/ Fungal/ Parasitic/ Protozoan Infections | “bacterial” OR “infection” OR “viral” OR “fungal” |
