# Supplementary material for: Host Biomarkers for Distinguishing Bacterial from Non-Bacterial Causes of Acute Febrile Illness: A Comprehensive Review
Source: PLoS One. 2016 Aug 3;11(8):e0160278. doi: 10.1371/journal.pone.0160278 (PMC4972355; doi:10.1371/journal.pone.0160278)
Supplement: S2 Table — (DOCX) [file pone.0160278.s004.docx]

| Criteria | Description | Issue Addressed | Source |
| --- | --- | --- | --- |
| 1a. Multi-center | Was the spectrum of patients representative of the patients who will receive the test in practice - Not a convenience sample at a single center? | Generalizability | QUADAS |
| 1b. Multi-severity | Was the spectrum of patients representative of the patients who will receive the test in practice - All severities? | Generalizability | QUADAS |
| 1c. Includes Adult & Pediatric | Was the spectrum of patients representative of the patients who will receive the test in practice - Includes Adults & Pediatric? | Generalizability | QUADAS |
| 1d. Prospective* | Was the disease state of the patients not known and obtained prospectively? | Selection bias | QUADAS & Lijmer criteria |
| 1d. Spectrum of bacterial infection types | Was the spectrum of patients representative of the patients who will receive the test in practice - Multiple bacterial pathogens/strains part of the analysis? | Generalizability | Custom |
| 1e. Includes infections affecting multiple body systems | Was the spectrum of patients representative of the patients who will receive the test in practice - Not just gastroenteritis, or meningitis, or pneumonia, etc.? | Generalizability | Custom |
| 2. Demographic information provided | Are data on patient age and gender provided? | Generalizability | Lijmer criteria |
| 3. Clinical setting described | Enough information to identify setting (community through tertiary care)? | Generalizability, Validity, Selection bias | Lijmer criteria |
| 4. Sample size ≥100 | Does the study population have at least 100 patients? | Generalizability, Validity, Accuracy | Custom |
| 5. Selection criteria defined | Were selection (inclusion/exclusion) criteria clearly described? | Generalizability, Validity | QUADAS |
| 6. Selection bias avoidance* | Were patients consecutively recruited? | Selection bias | Lijmer criteria |
| 7. Duration of illness mentioned | Was the duration of illness (e.g., symptoms, fever, length of hospital stay) mentioned? | Generalizability, Validity | Lijmer criteria |
| 8. Reference test correctly classifies | Is the reference test likely to correctly classify the target condition? | Misclassification | QUADAS |
| 9. Testing within viable time-frame* | Is the time period between reference standard and index test short enough to be reasonably sure that the target condition did not change between the two tests? | Misclassification | QUADAS & Lijmer criteria |
| 10. Reference testing on whole sample vs. subset | Was the whole study sample given the reference test? | Selection bias | QUADAS |
| 11. All had same reference (per study group) | Did patients receive the same reference test regardless of the index test result? | Differential verification bias | QUADAS |
| 12. Treatment paradox avoidance | Measurement of reference standard before any interventions were started, without knowledge of test results? | Variability, Affects accuracy of quantitative estimate of diagnostic performance | Lijmer criteria |
| 13. Reference test independent of index (biomarker) test | Was the reference standard independent of the index test (i.e. the index test did not form part of the reference standard)? | Incorporation bias | QUADAS |
| 14. Index (biomarker) test methodology described | Was the execution of the index (biomarker) test described in sufficient detail to permit replication of the test? | Variability | QUADAS |
| 15. Reference test methodology described | Was the execution of the reference test described in sufficient detail to permit its replication? | Variability | QUADAS |
| 16. Index (biomarker) test interpret without reference test results* | Were the index (biomarker) test results interpreted without knowledge of the results of the reference test? | Blinding | QUADAS & Lijmer criteria |
| 17. Reference test interpret without index test results* | Were the reference test results interpreted without knowledge of the results of the index test? | Review bias (inflates diagnostic accuracy) | QUADAS & Lijmer criteria |
| 18. Real-world interpretation | Were the same clinical data available when test results were interpreted as would be available when the test is used in practice? | Affects accuracy of quantitative estimate of diagnostic performance | QUADAS |
| 19. Un-interpretable/ intermediate results | Were un-interpretable/ intermediate test results reported? | Affects accuracy of quantitative estimate of diagnostic performance | QUADAS |
| 20. Withdrawals explained | Were withdrawals from the study explained? | Affects accuracy of quantitative estimate of diagnostic performance | QUADAS |
| 21. Reported Diagnostic accuracy results | Do the authors report sensitivity and specificity values? | Required for assessing diagnostic accuracy | Custom |

** Criteria that overlap between QUADAS and Lijmer et al. (1999).*
